# Supplementary figures and images for: The Effect of Super-Repressor IkB-Loaded Exosomes (Exo-srIκBs) in Chronic Post-Ischemia Pain (CPIP) Models
Source: Pharmaceutics. 2023 Feb 7;15(2):553. doi: 10.3390/pharmaceutics15020553 (PMC9958867; doi:10.3390/pharmaceutics15020553)

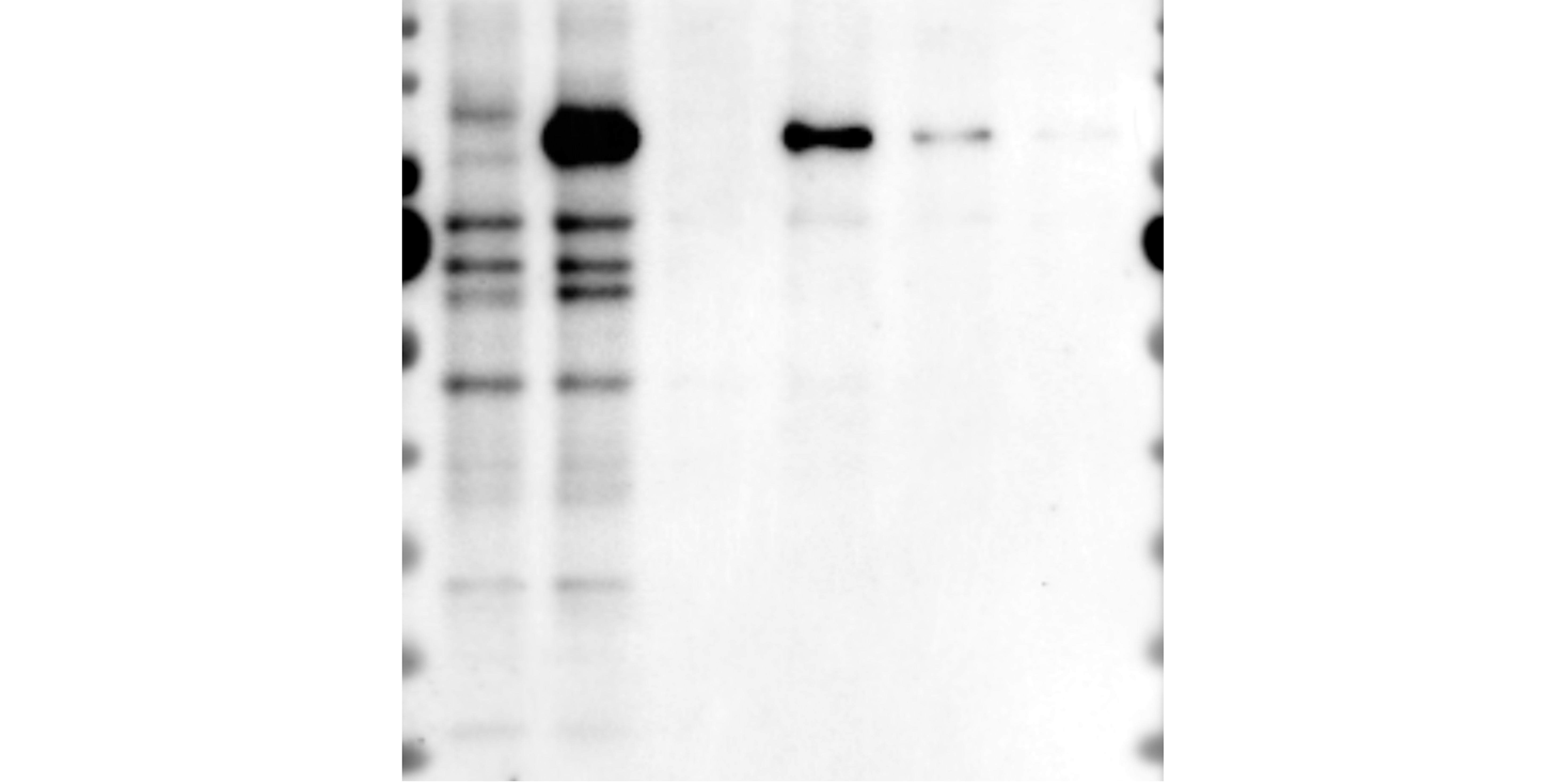

Supplement: Supplementary file 1 [file pharmaceutics-15-00553-s001.zip › Figure S1/1) srIkb.tif]

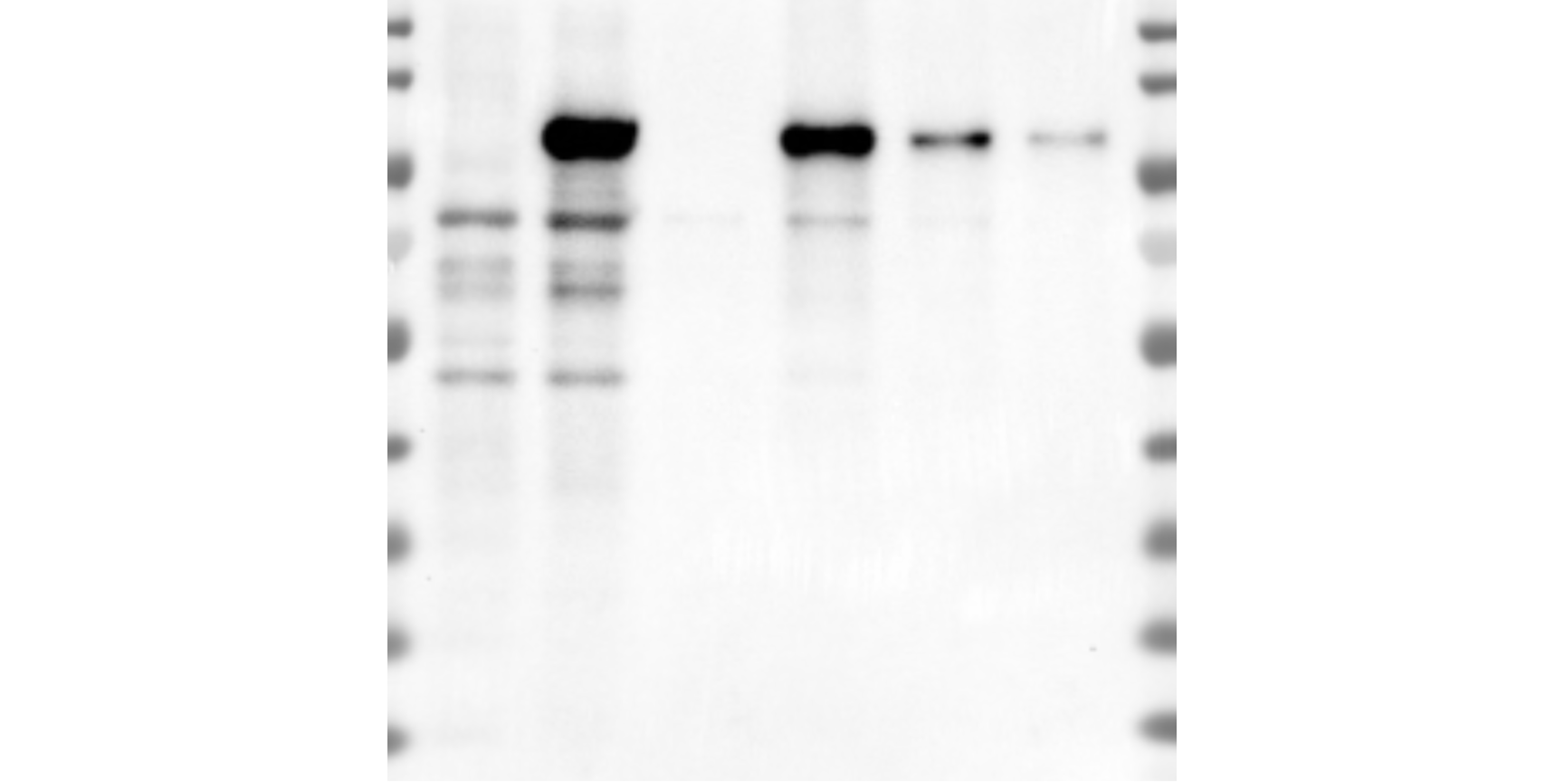

Supplement: Supplementary file 1 [file pharmaceutics-15-00553-s001.zip › Figure S1/2) CRY2.tif]

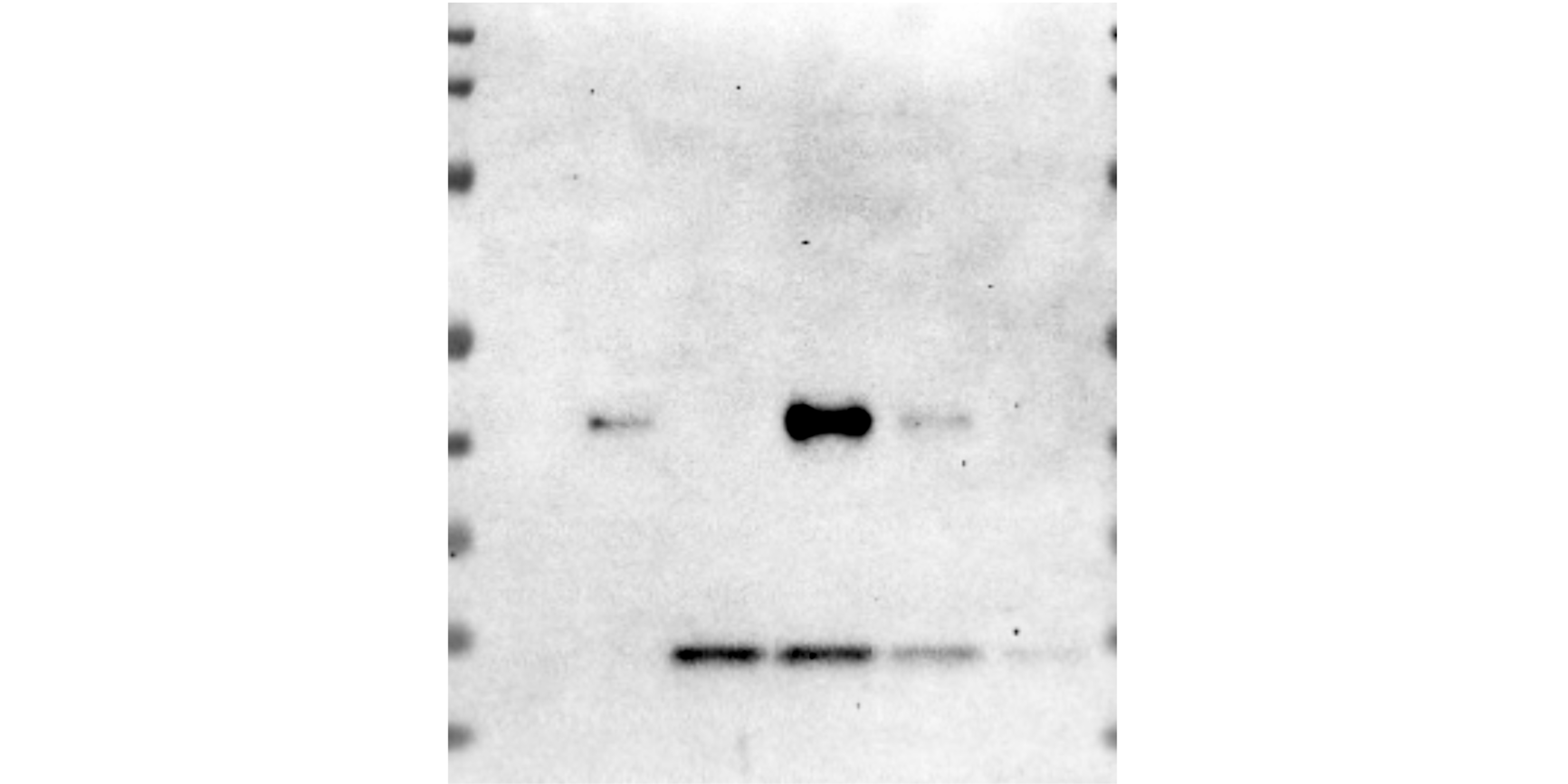

Supplement: Supplementary file 1 [file pharmaceutics-15-00553-s001.zip › Figure S1/3) CD9.tif]

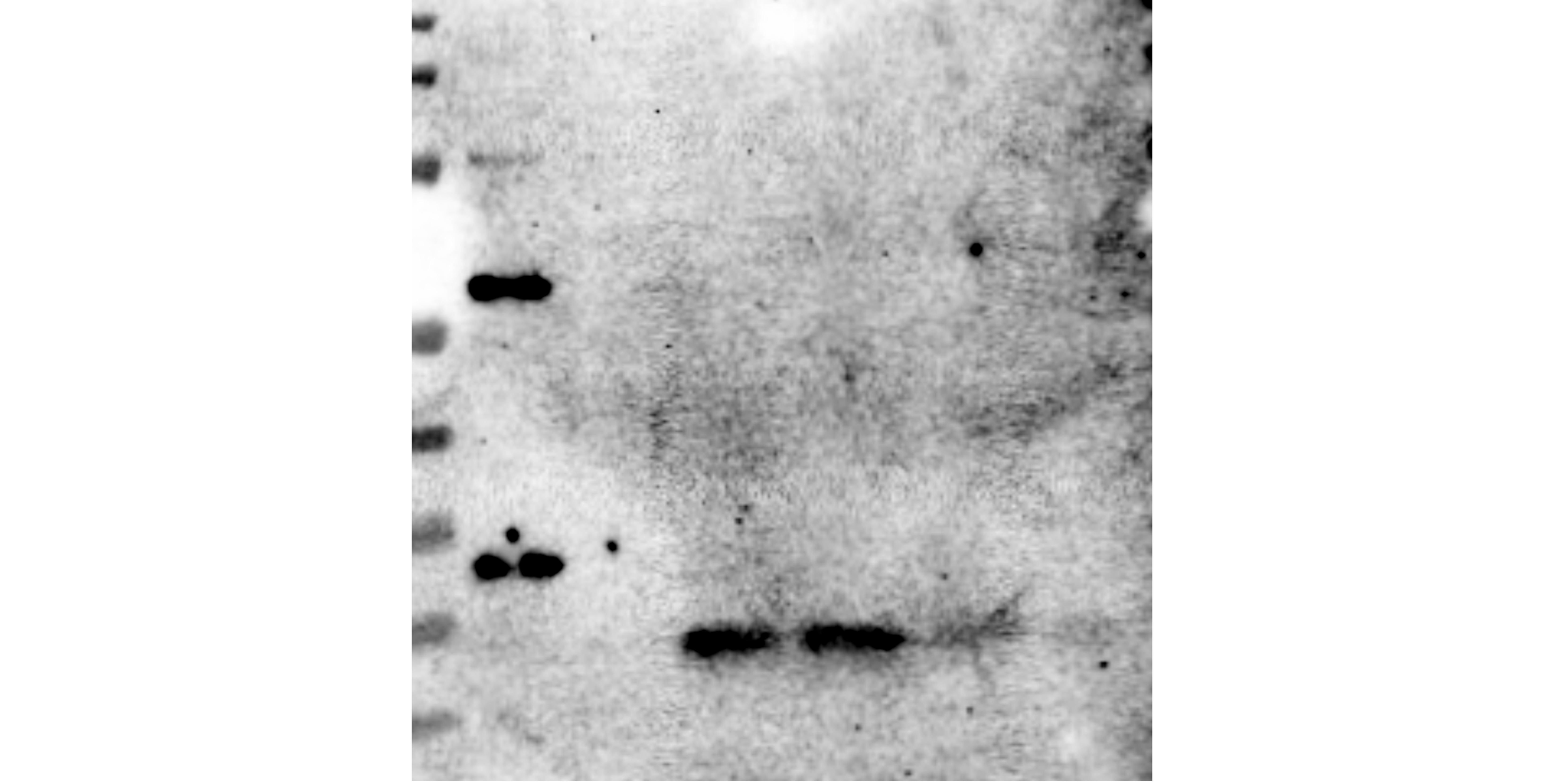

Supplement: Supplementary file 1 [file pharmaceutics-15-00553-s001.zip › Figure S1/4) CD81.tif]

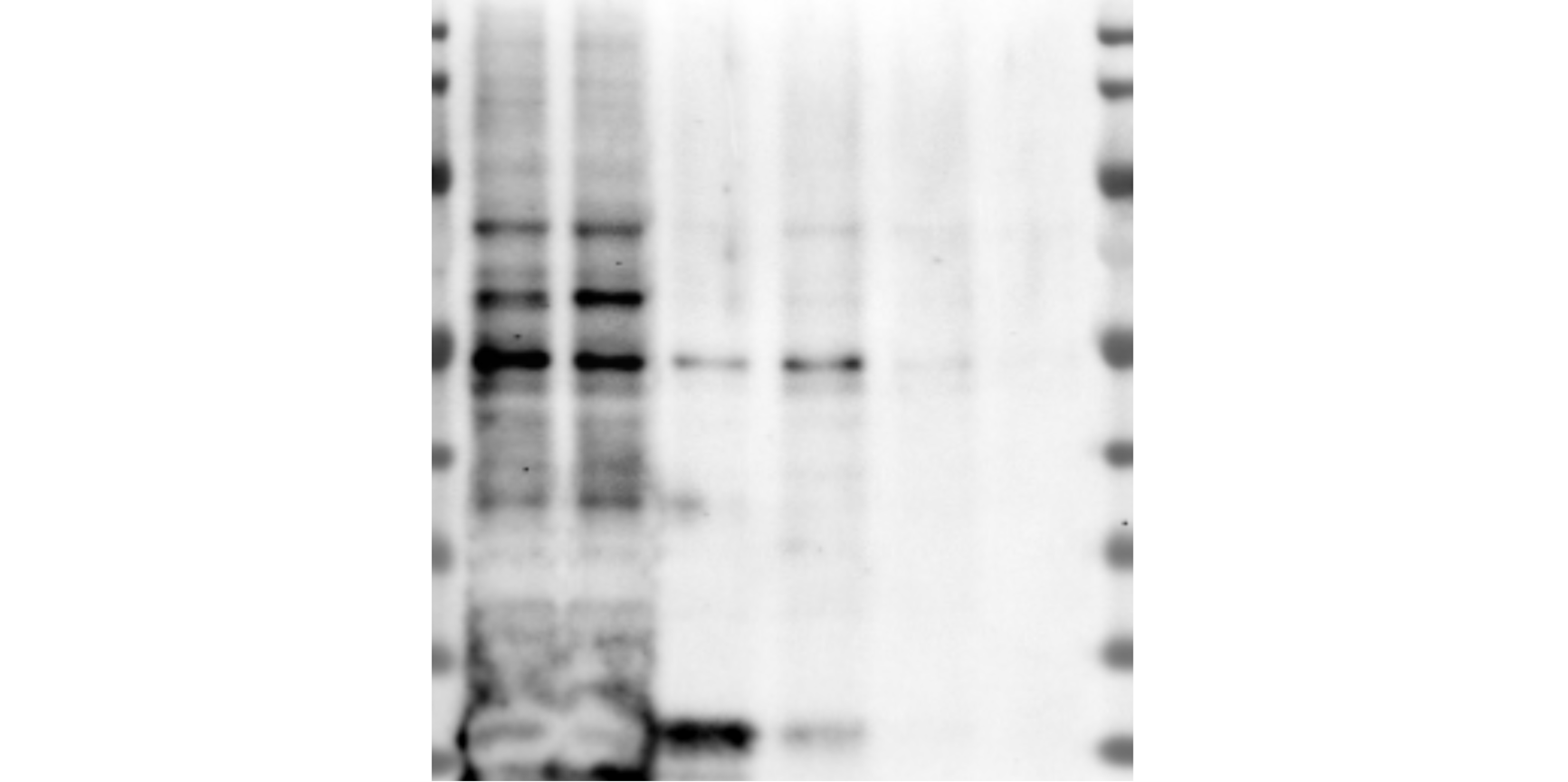

Supplement: Supplementary file 1 [file pharmaceutics-15-00553-s001.zip › Figure S1/5) TSG101.tif]

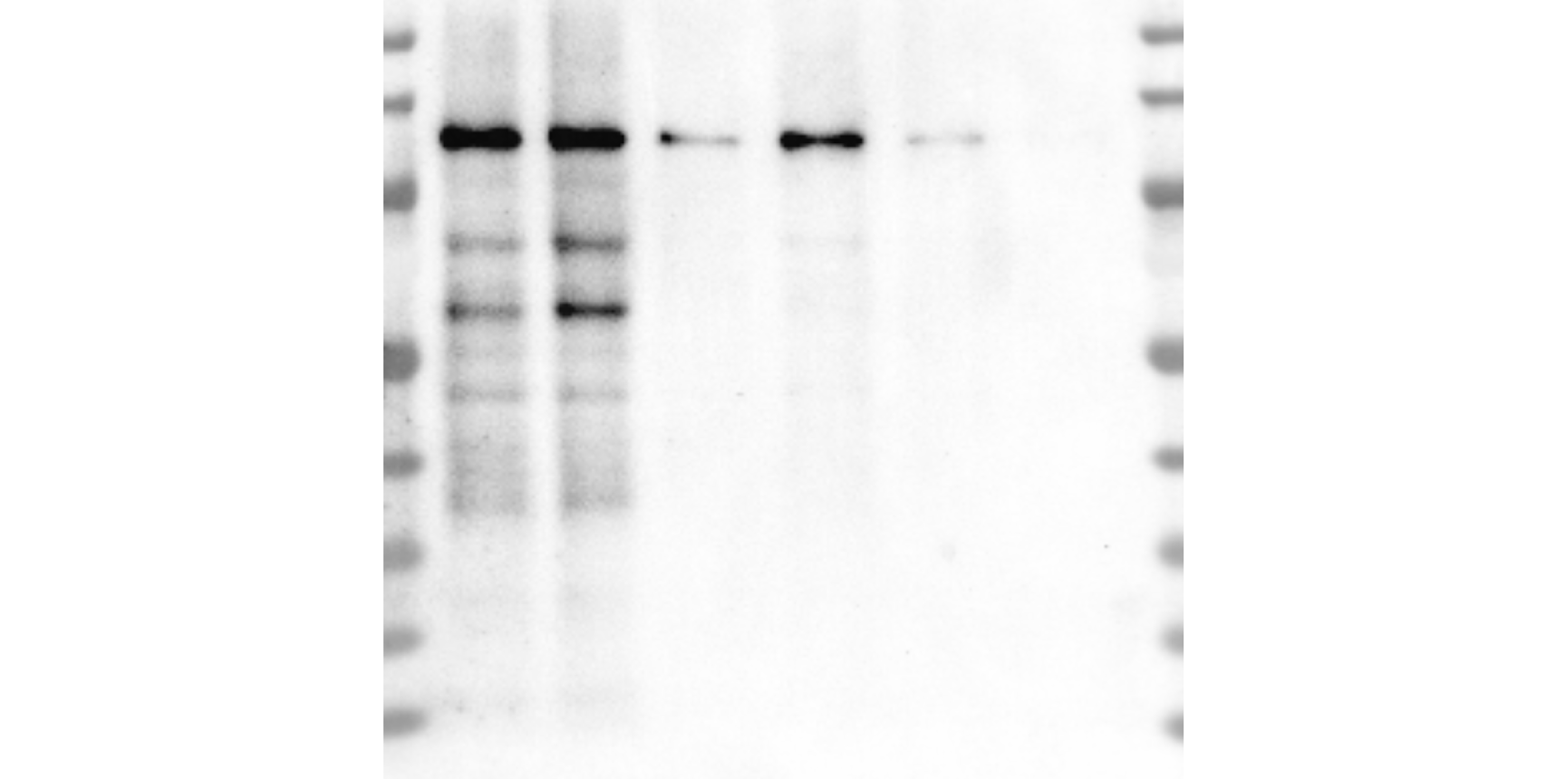

Supplement: Supplementary file 1 [file pharmaceutics-15-00553-s001.zip › Figure S1/6) Alix.tif]

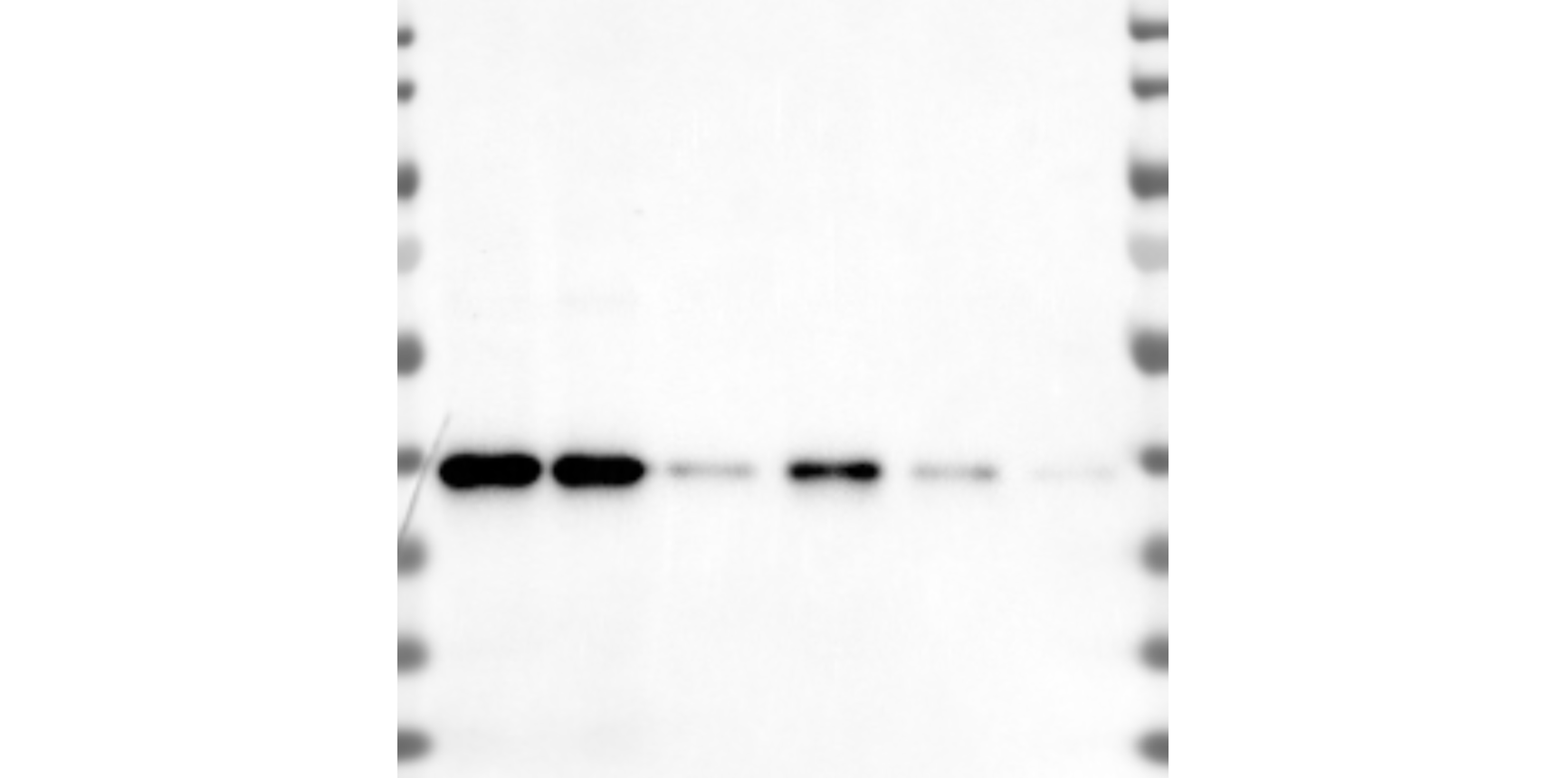

Supplement: Supplementary file 1 [file pharmaceutics-15-00553-s001.zip › Figure S1/7) GAPDH.tif]

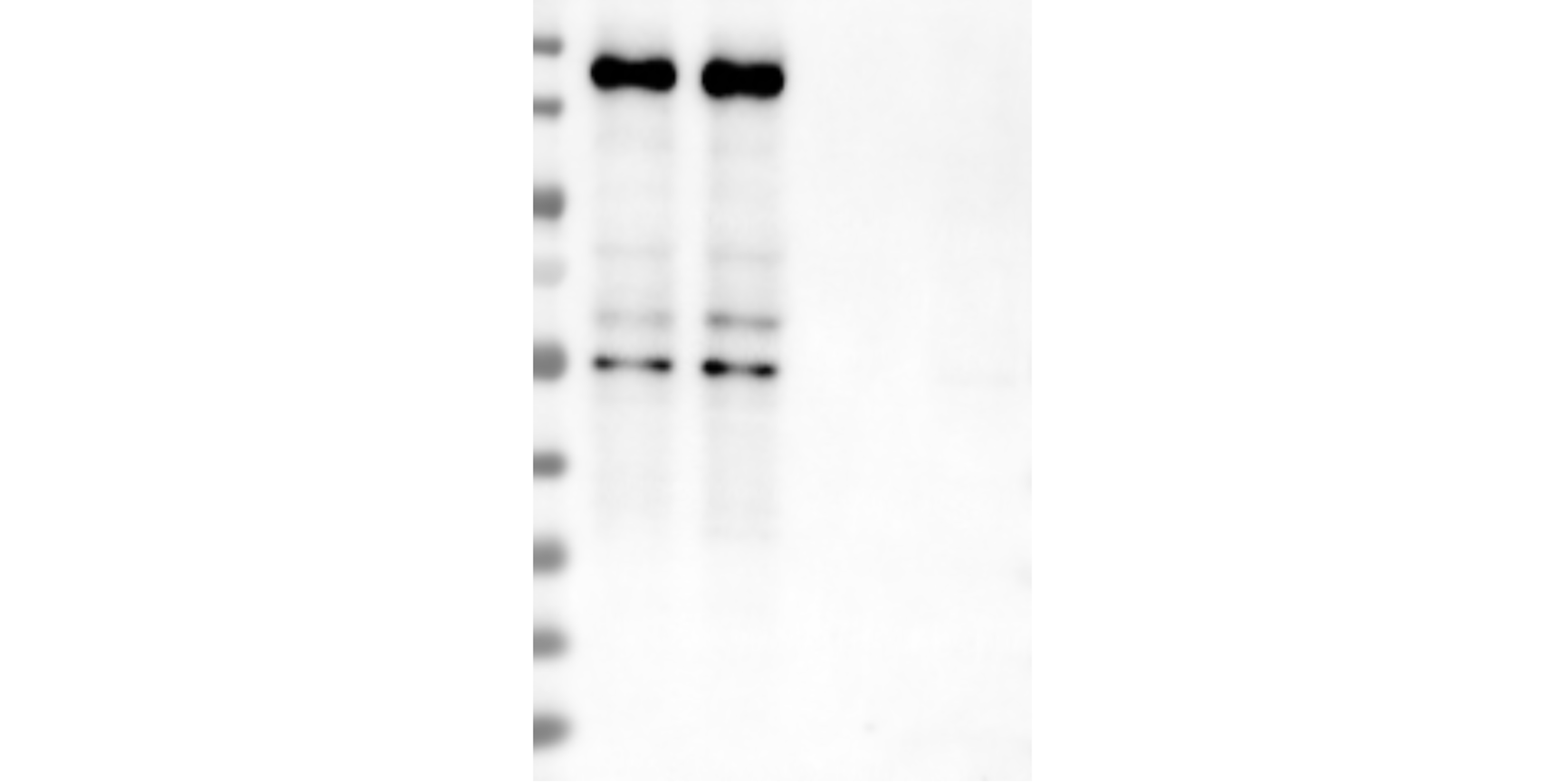

Supplement: Supplementary file 1 [file pharmaceutics-15-00553-s001.zip › Figure S2/1) GM130.tif]

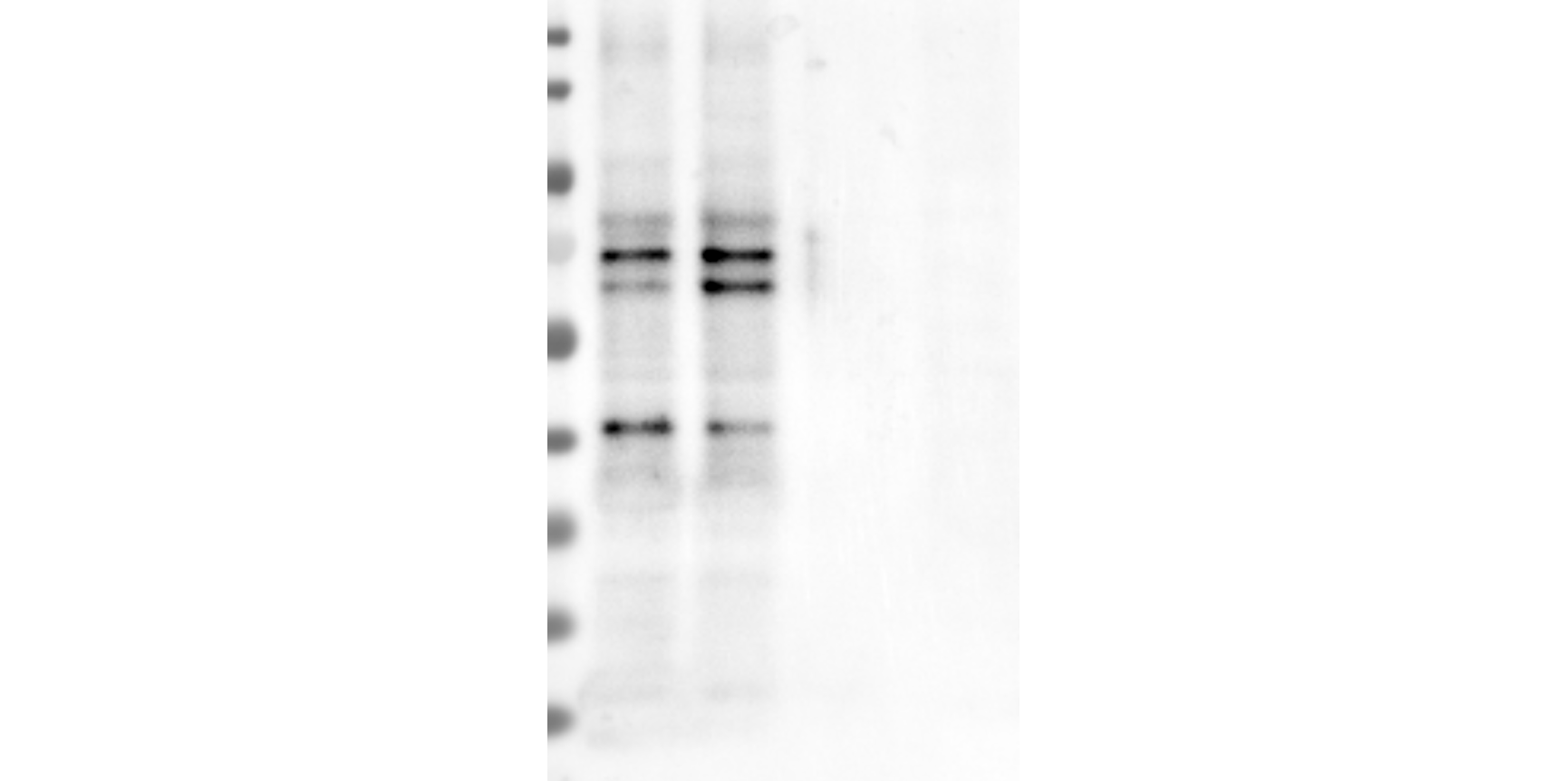

Supplement: Supplementary file 1 [file pharmaceutics-15-00553-s001.zip › Figure S2/2) LaminB1.tif]

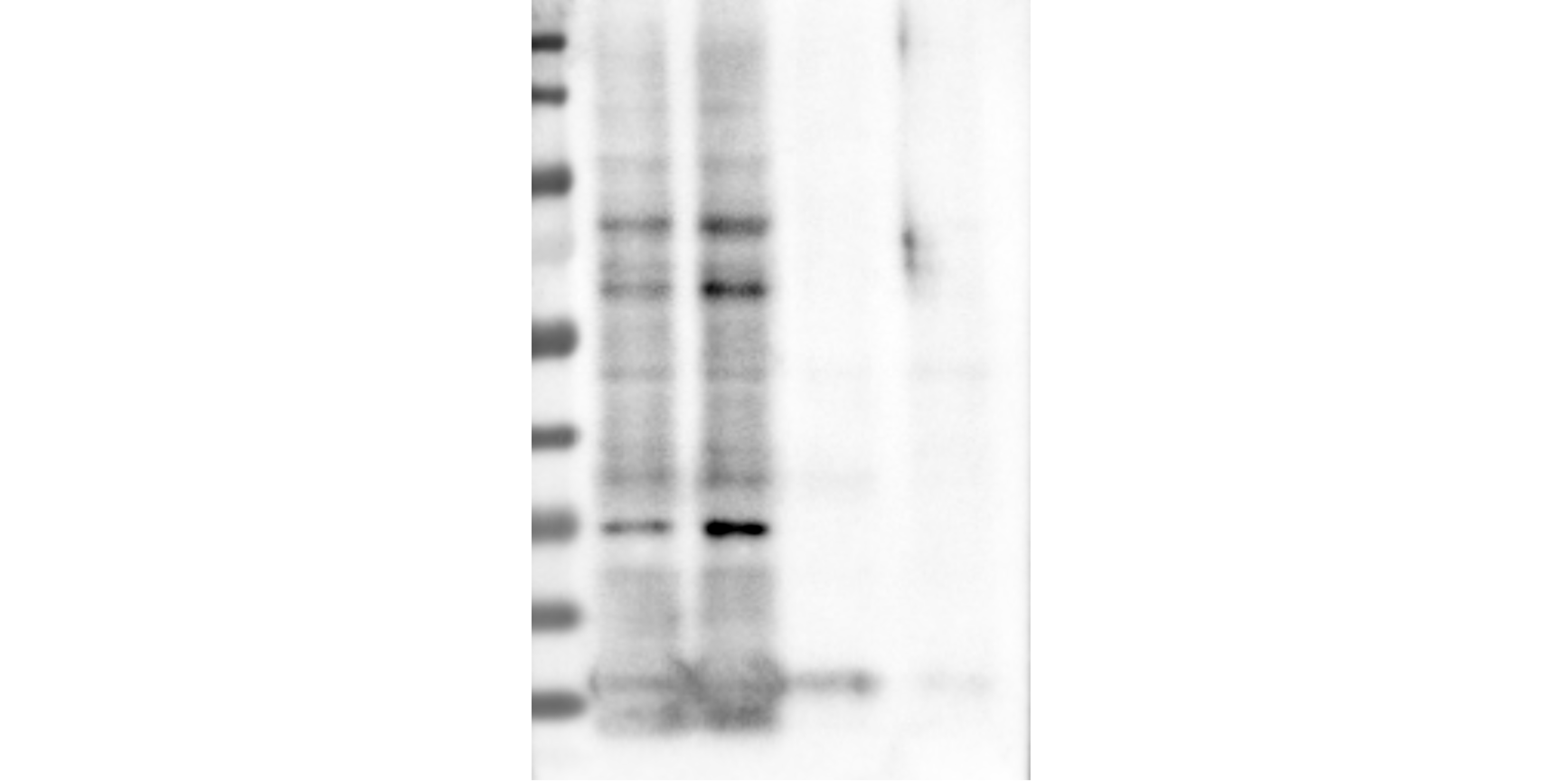

Supplement: Supplementary file 1 [file pharmaceutics-15-00553-s001.zip › Figure S2/3) Prohibitin.tif]

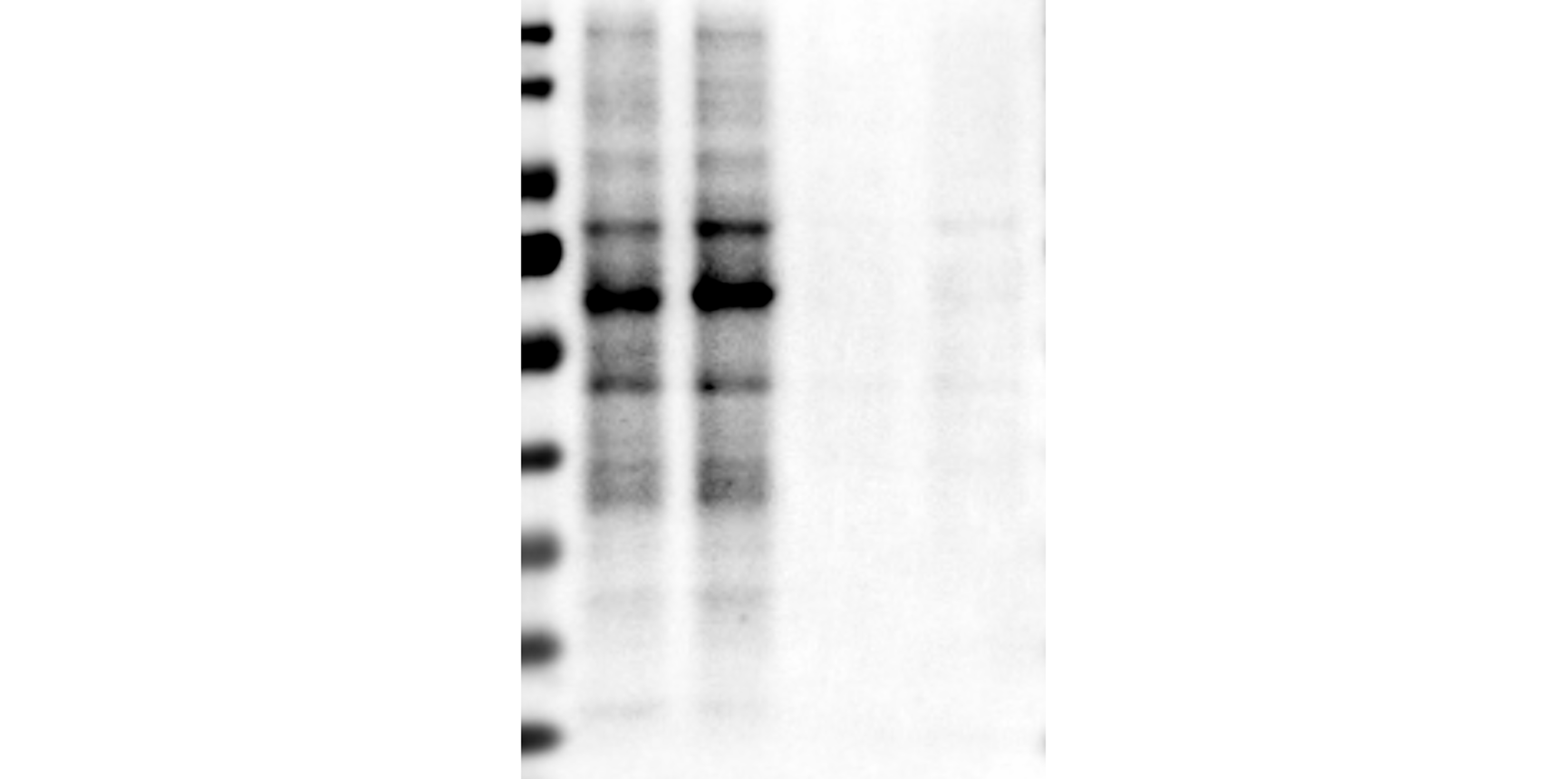

Supplement: Supplementary file 1 [file pharmaceutics-15-00553-s001.zip › Figure S2/4) Calnexin.tif]

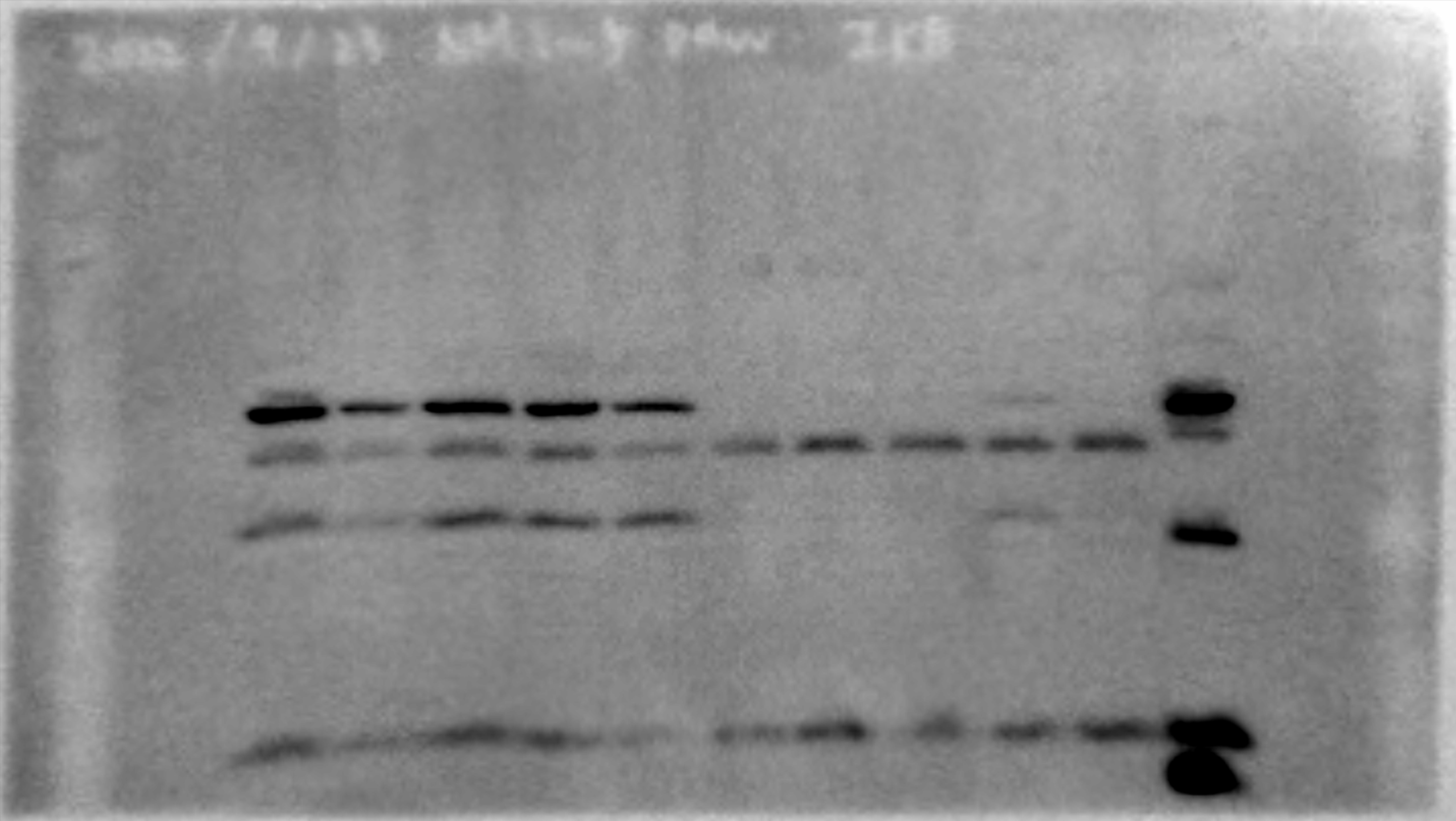

Supplement: Supplementary file 1 [file pharmaceutics-15-00553-s001.zip › Figure S3/Sham_CPIP_IkB.tif]

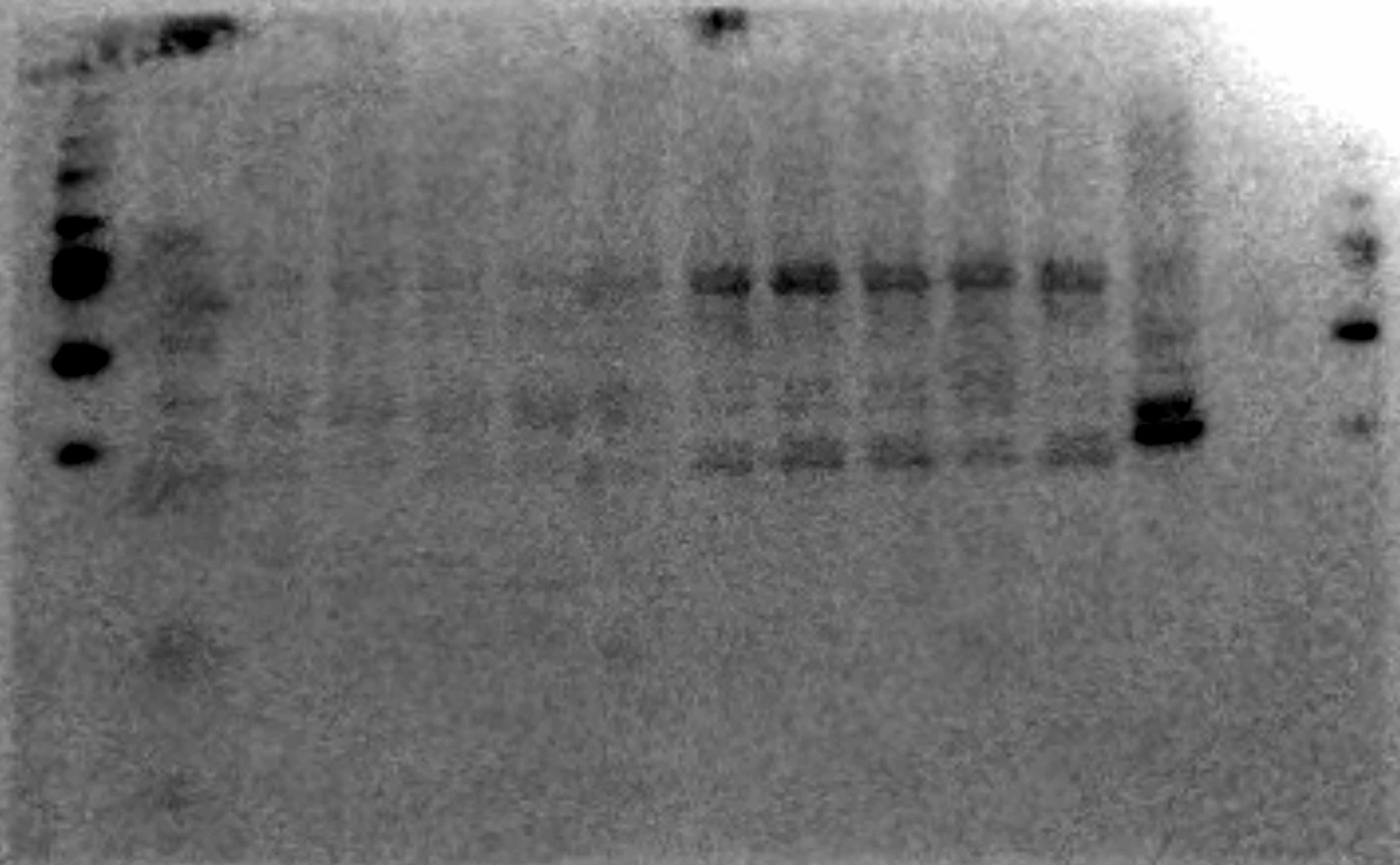

Supplement: Supplementary file 1 [file pharmaceutics-15-00553-s001.zip › Figure S3/Sham_CPIP_pIkB.tif]

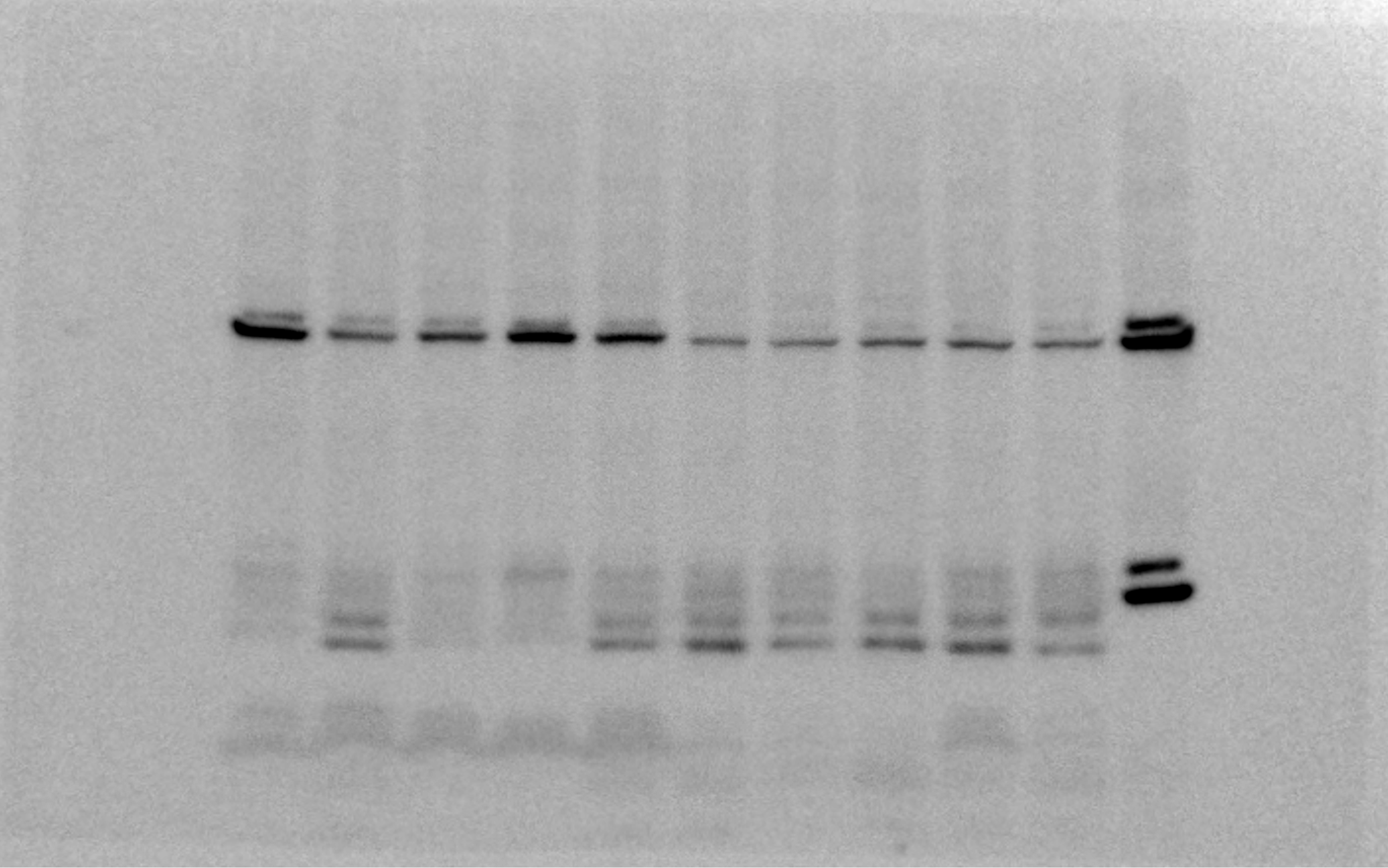

Supplement: Supplementary file 1 [file pharmaceutics-15-00553-s001.zip › Figure S3/Sham_CPIP_tubulin_.tif]

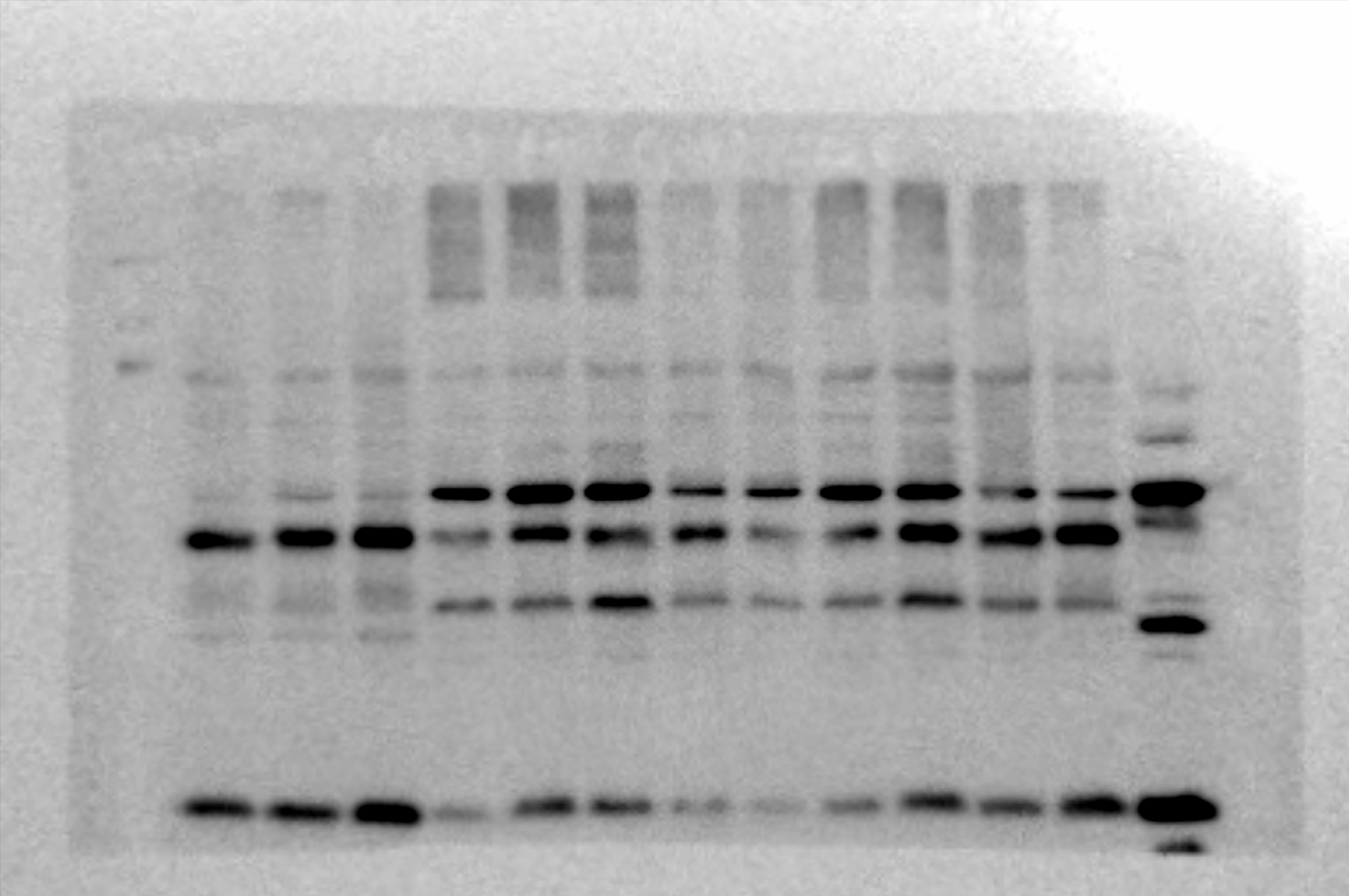

Supplement: Supplementary file 1 [file pharmaceutics-15-00553-s001.zip › Figure S4/24h after drug injection_IkB_1-3.tif]

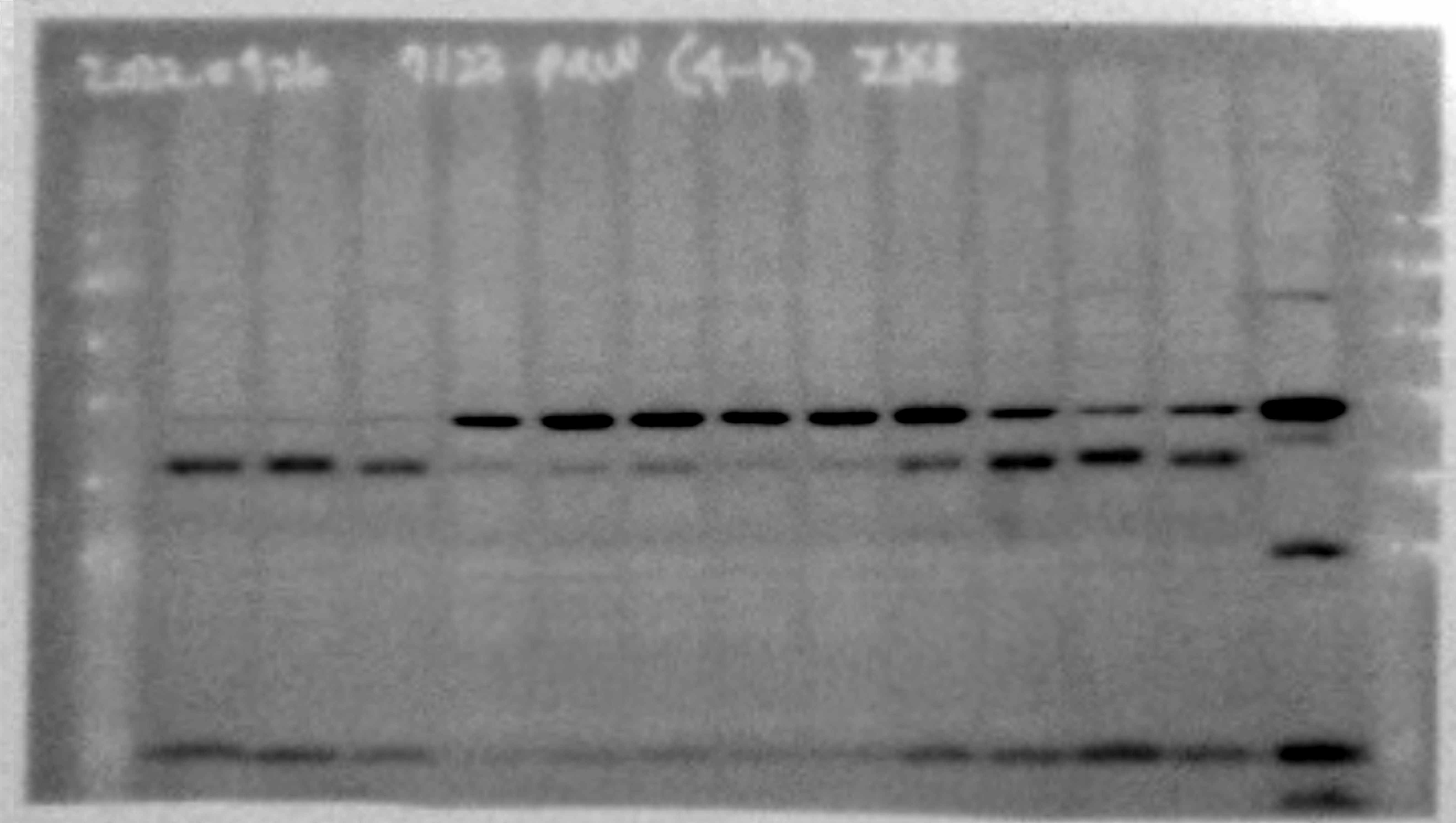

Supplement: Supplementary file 1 [file pharmaceutics-15-00553-s001.zip › Figure S4/24h after drug injection_IkB_4-6.tif]

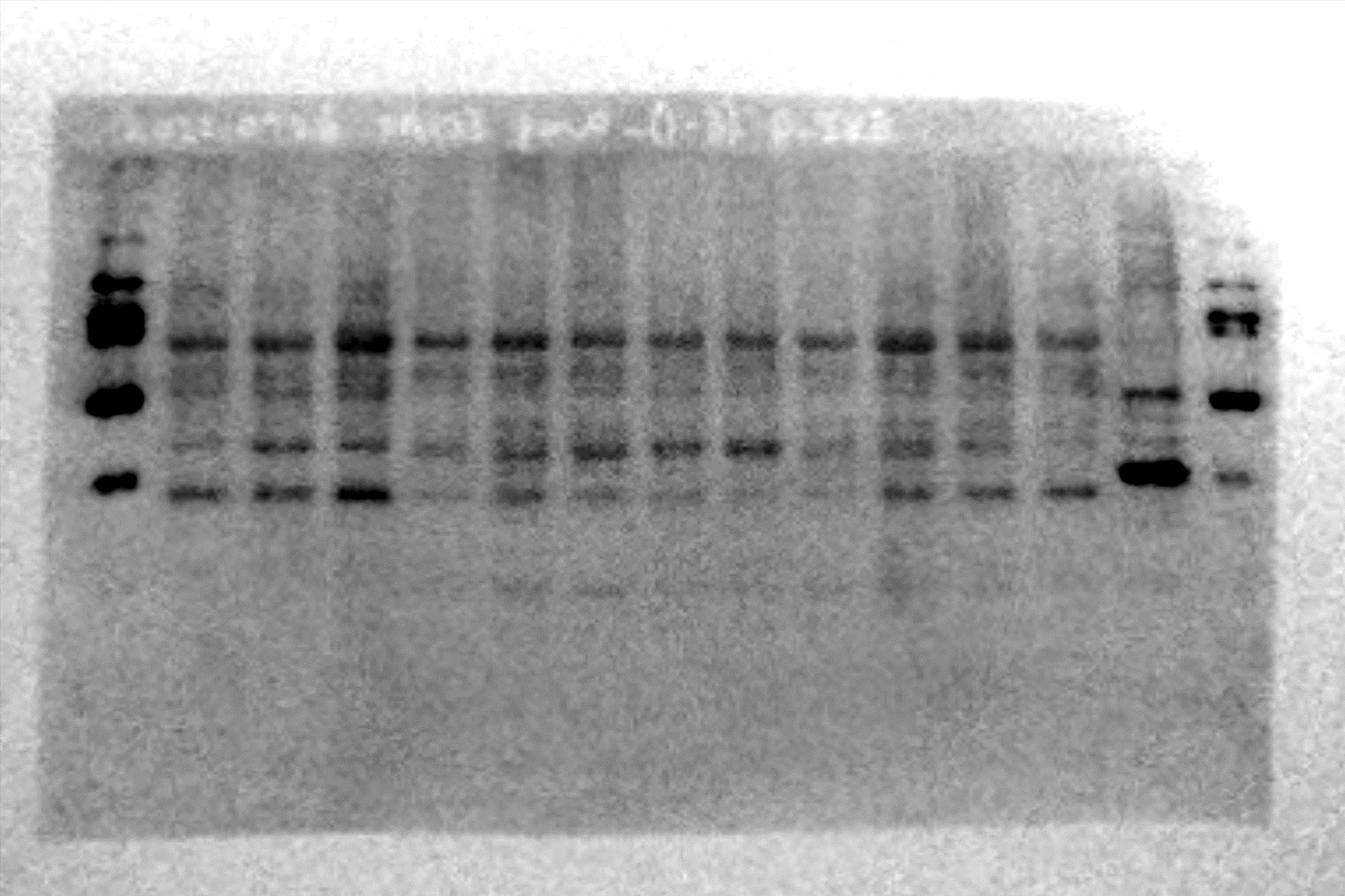

Supplement: Supplementary file 1 [file pharmaceutics-15-00553-s001.zip › Figure S4/24h after drug injection_pIkB_1-3.tif]

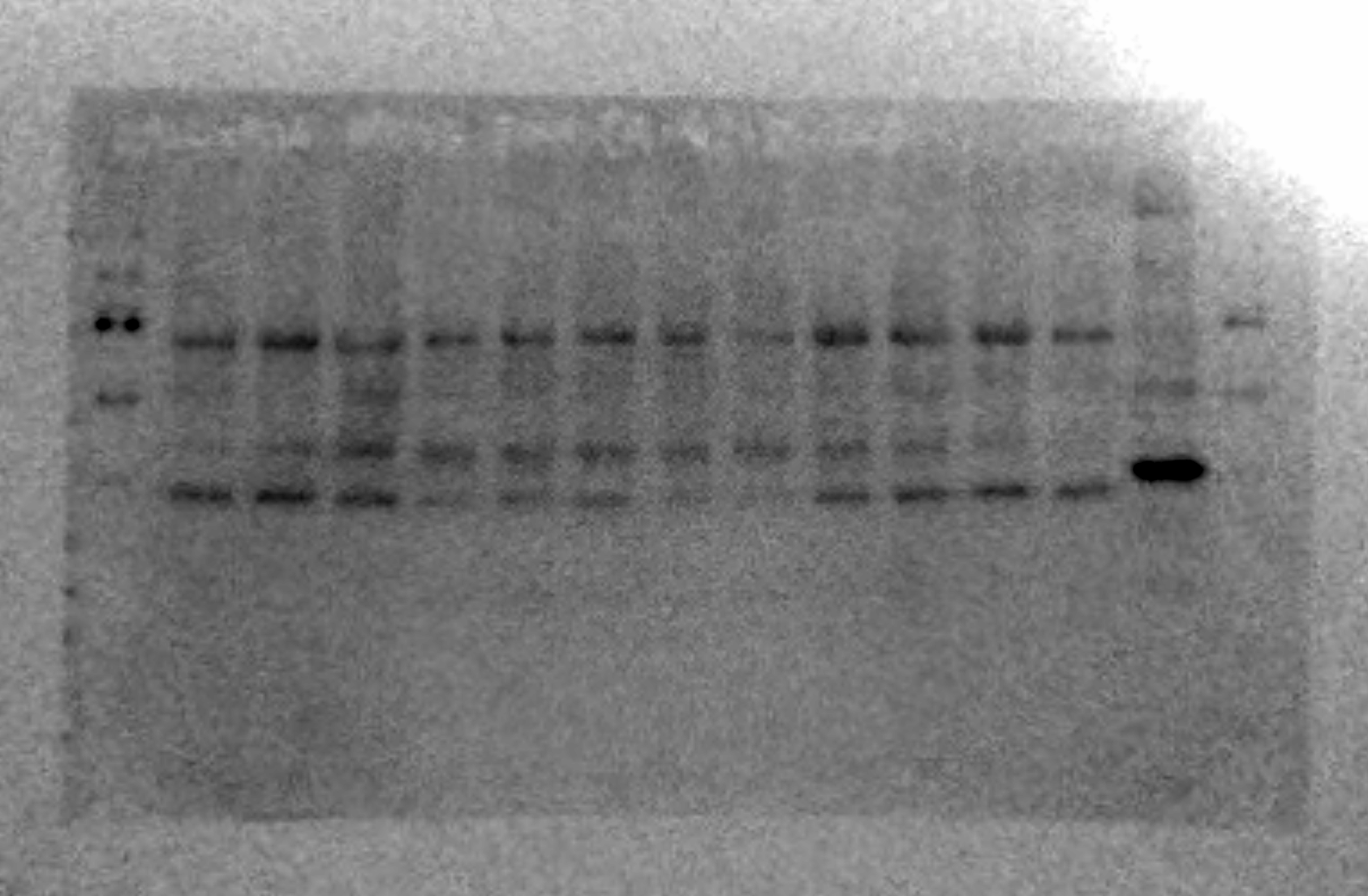

Supplement: Supplementary file 1 [file pharmaceutics-15-00553-s001.zip › Figure S4/24h after drug injection_pIkB_4-6.tif]

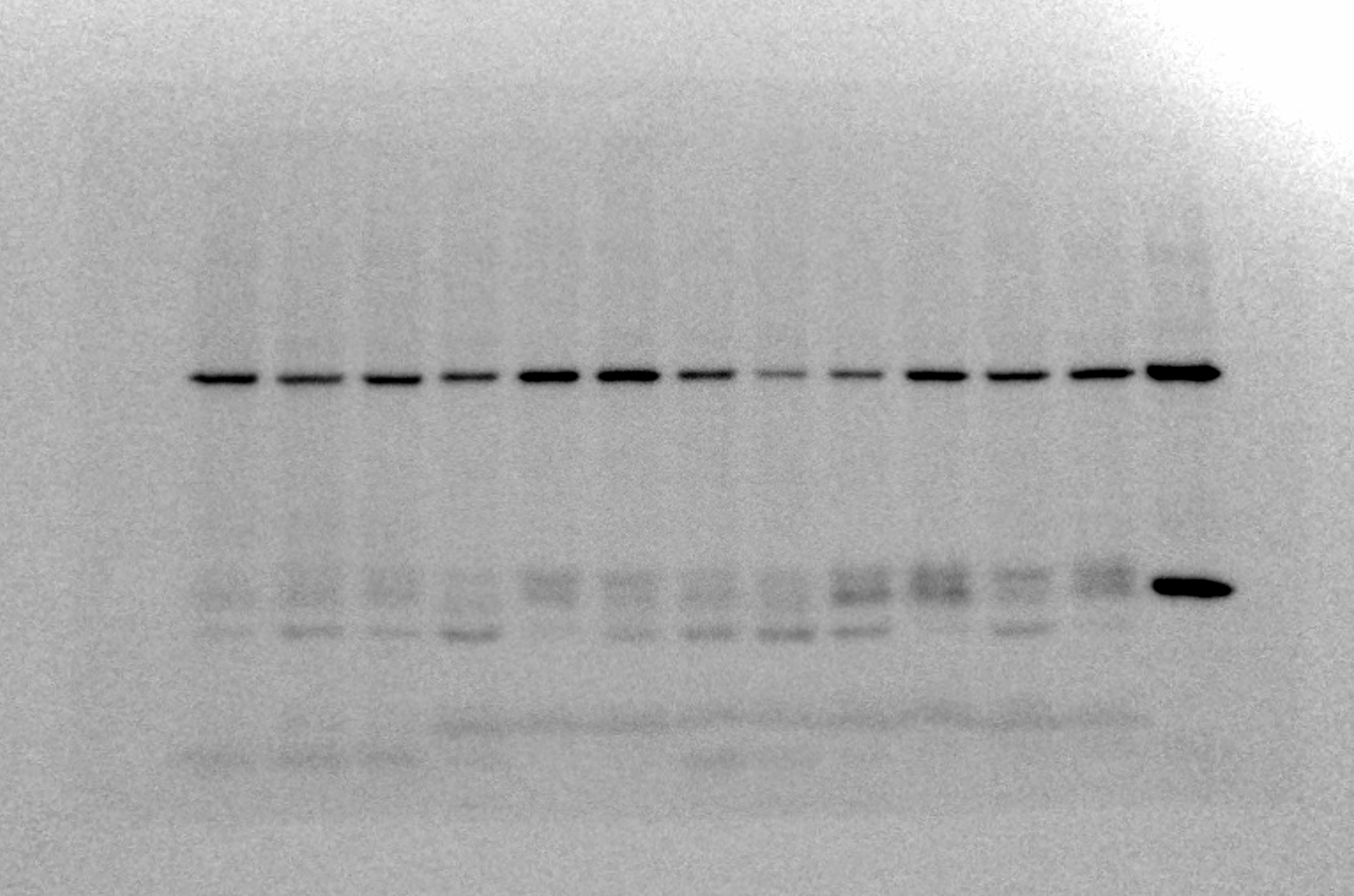

Supplement: Supplementary file 1 [file pharmaceutics-15-00553-s001.zip › Figure S4/24h after drug injection_tubulin_1-3.tif]

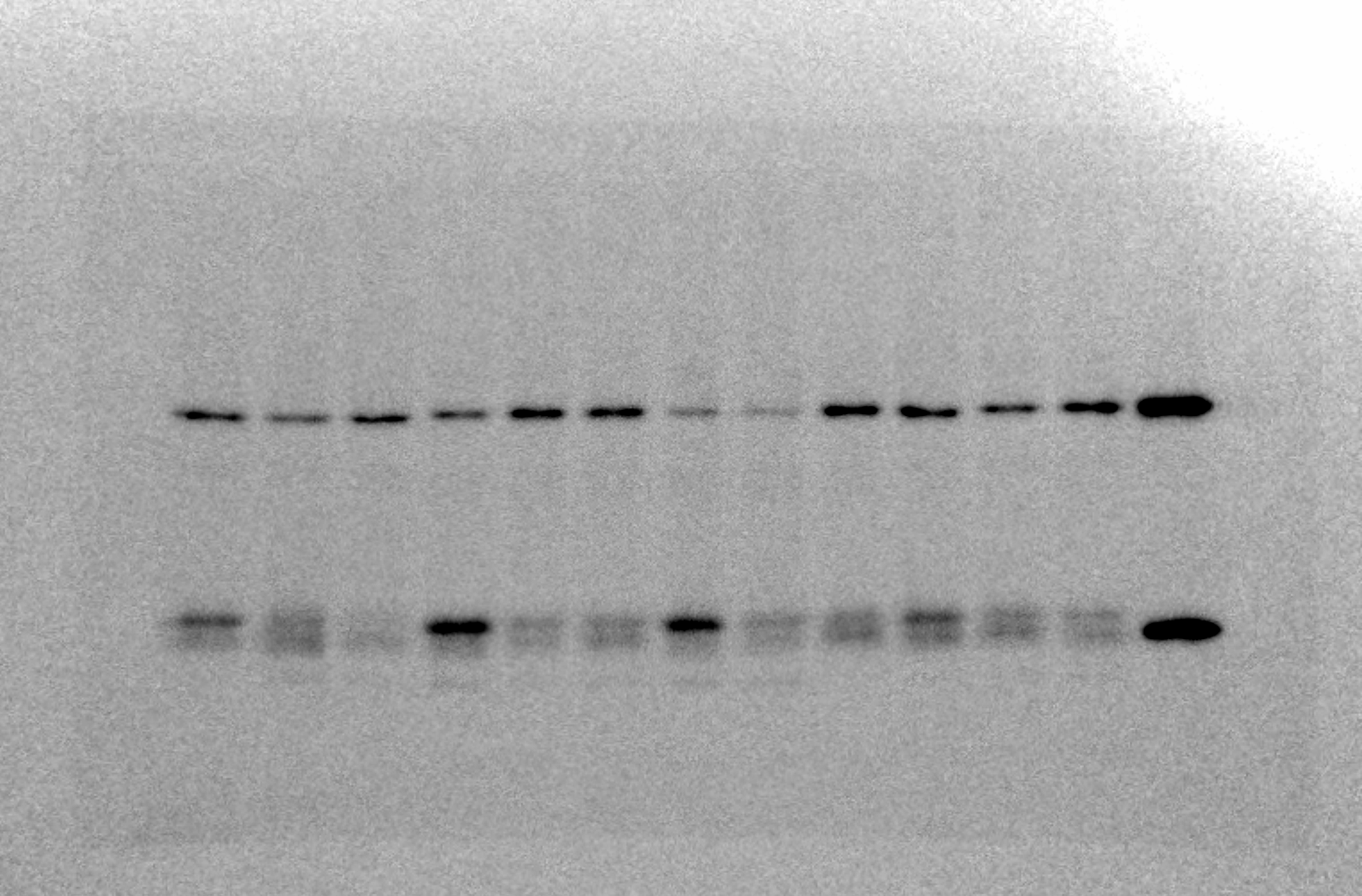

Supplement: Supplementary file 1 [file pharmaceutics-15-00553-s001.zip › Figure S4/24h after drug injection_tubulin_4-6.tif]

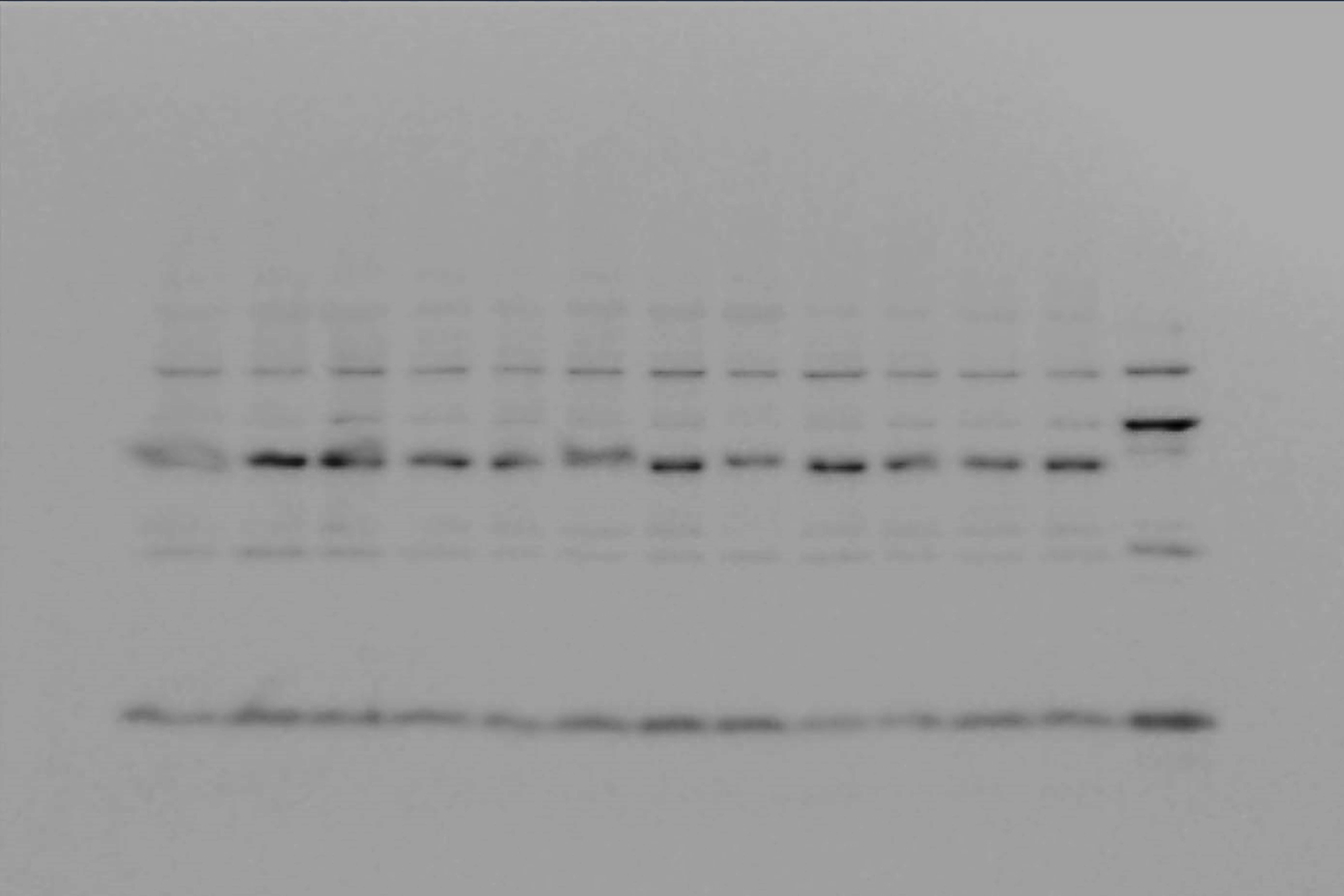

Supplement: Supplementary file 1 [file pharmaceutics-15-00553-s001.zip › Figure S5/48h after drug injection_IkB_1-3.tif]

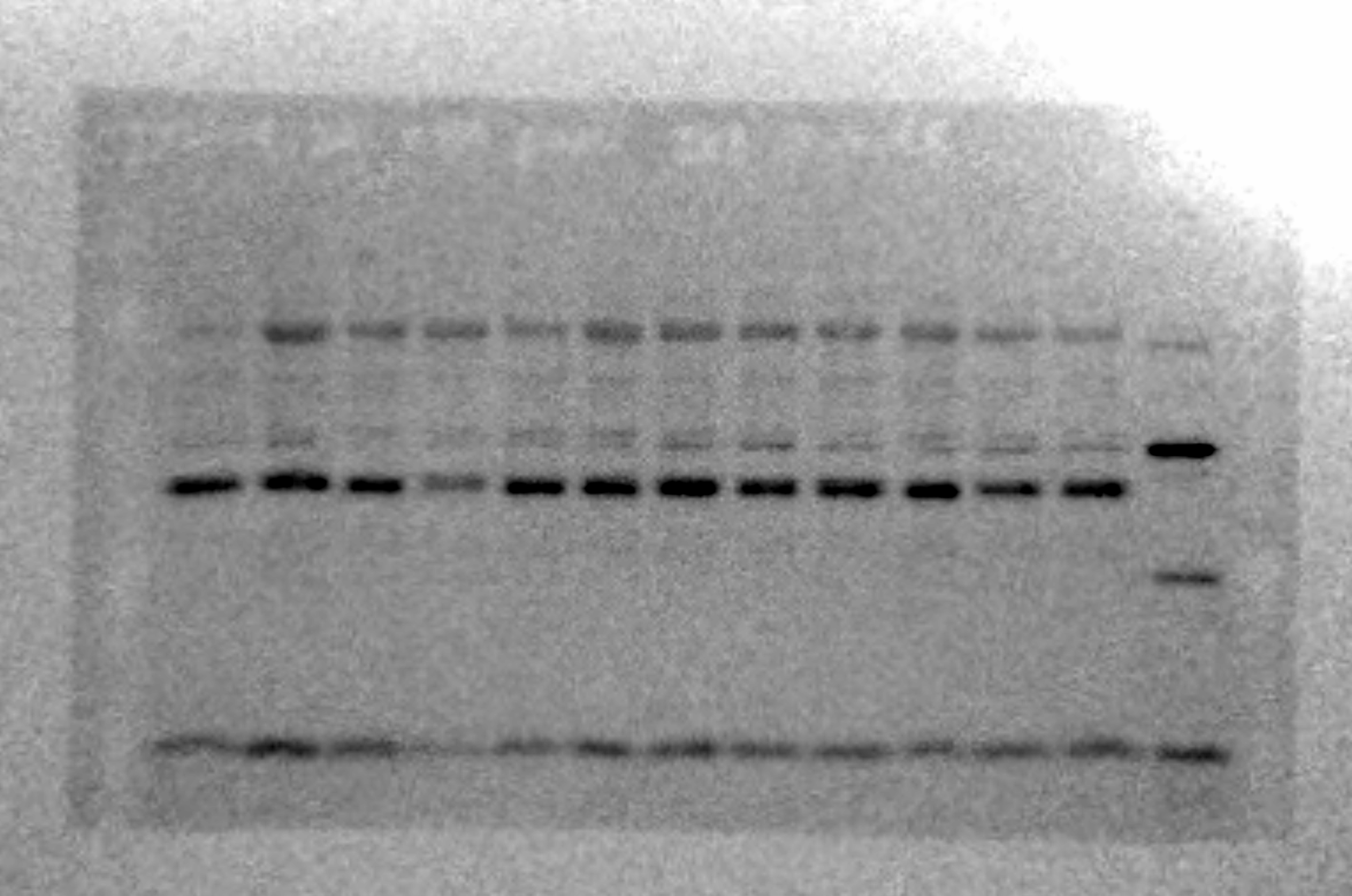

Supplement: Supplementary file 1 [file pharmaceutics-15-00553-s001.zip › Figure S5/48h after drug injection_IkB_4-6.tif]

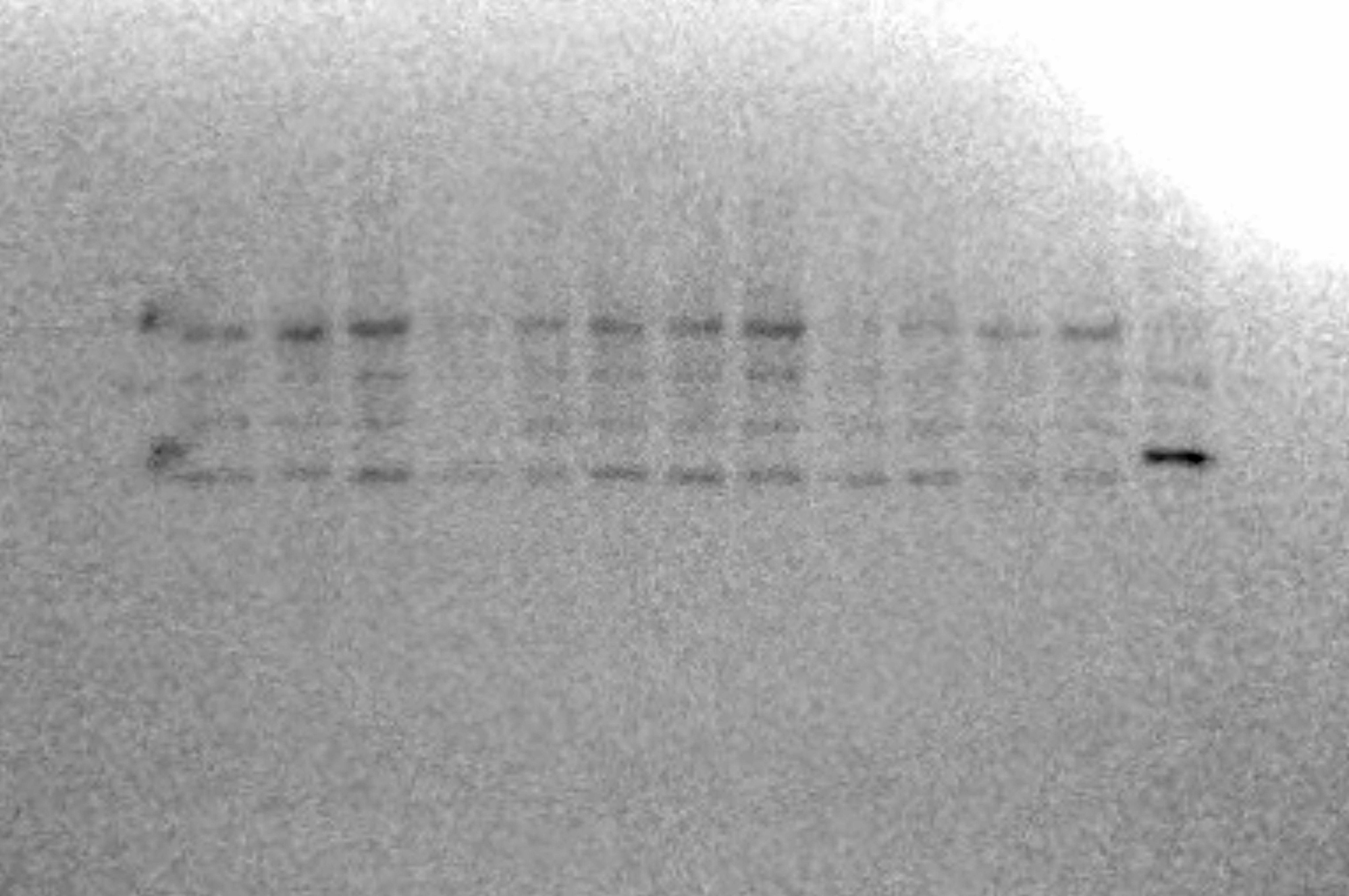

Supplement: Supplementary file 1 [file pharmaceutics-15-00553-s001.zip › Figure S5/48h after drug injection_pIkB_1-3.tif]

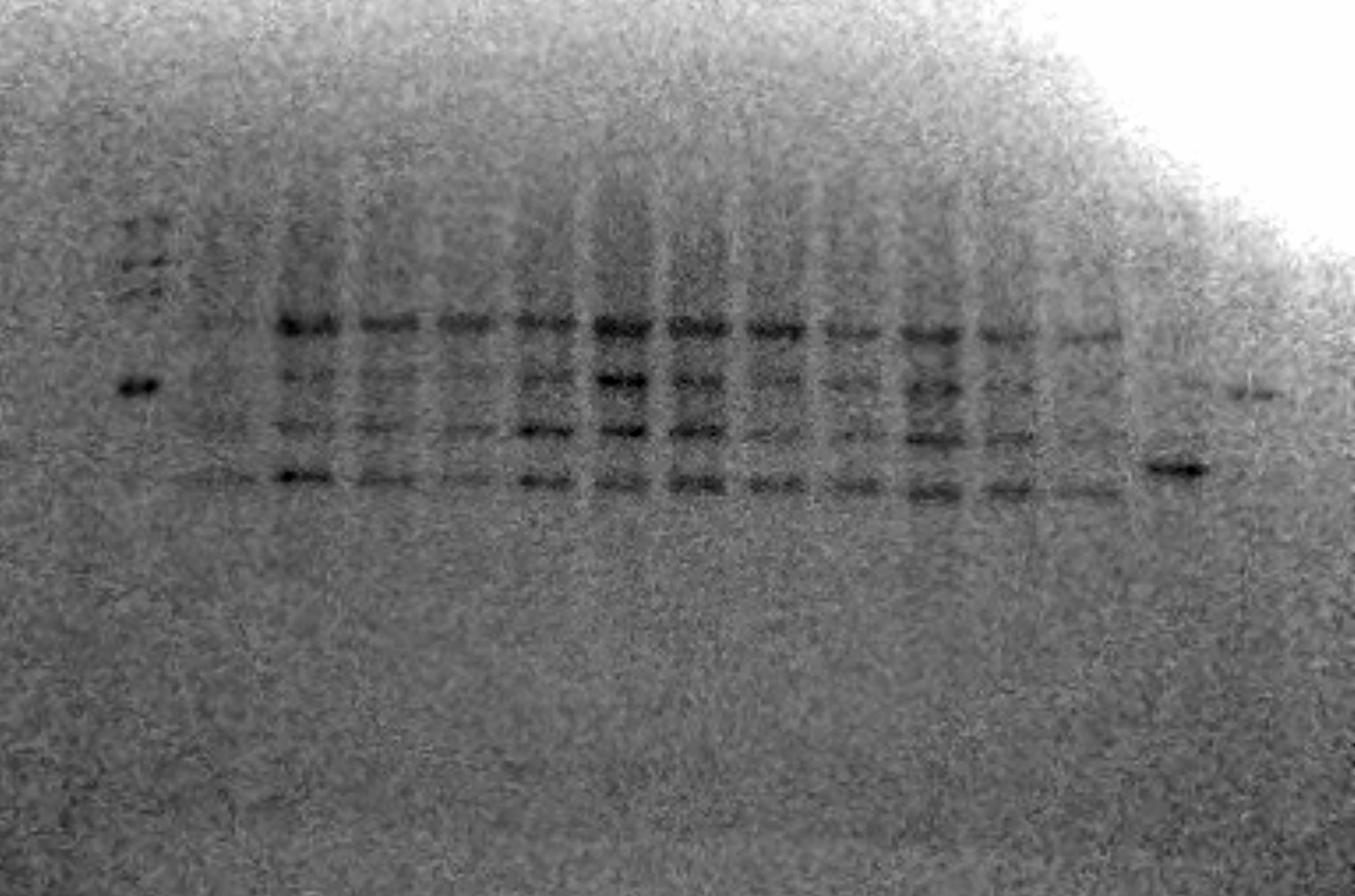

Supplement: Supplementary file 1 [file pharmaceutics-15-00553-s001.zip › Figure S5/48h after drug injection_pIkB_4-6.tif]

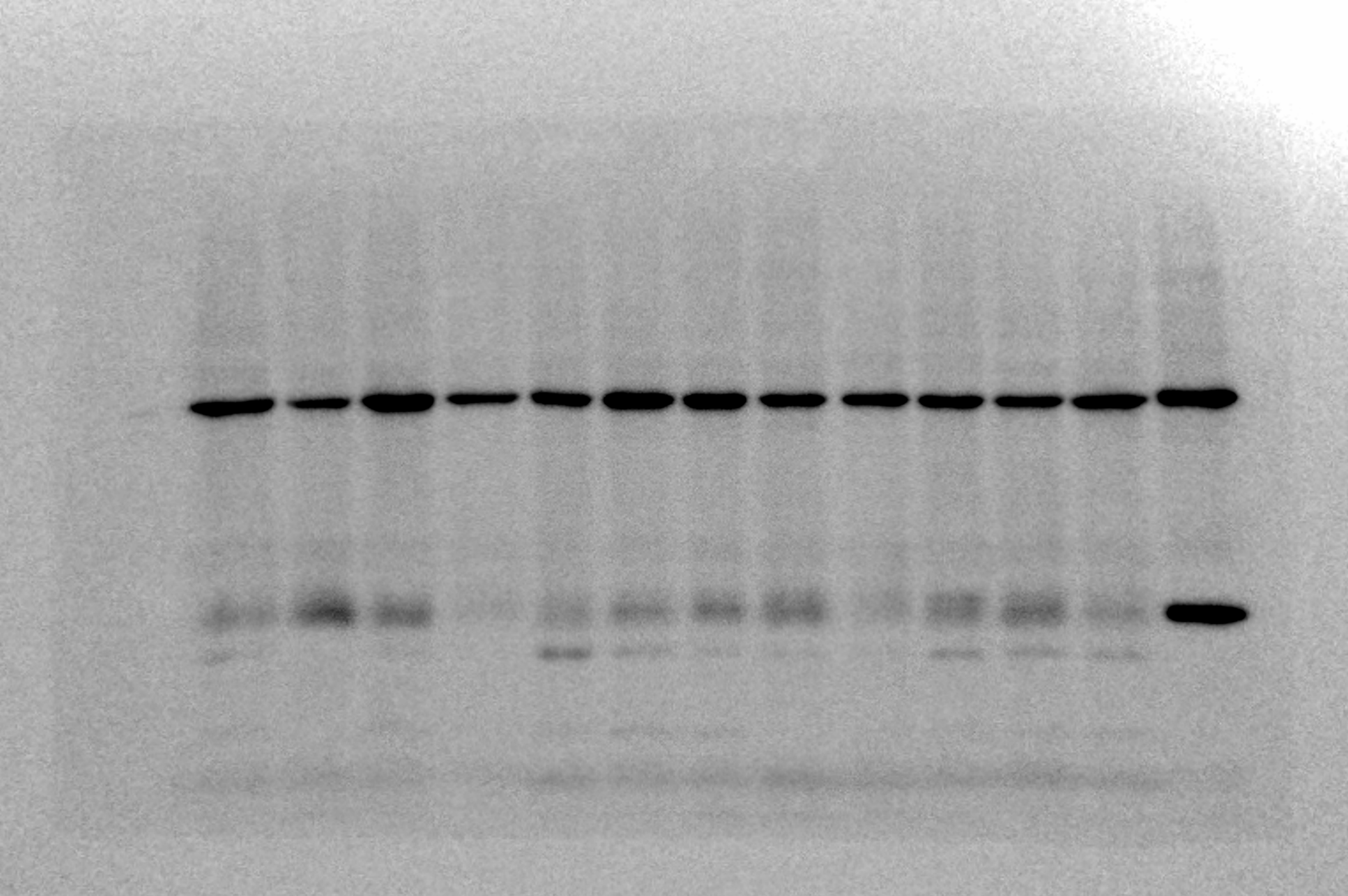

Supplement: Supplementary file 1 [file pharmaceutics-15-00553-s001.zip › Figure S5/48h after drug injection_tubulin_1-3.tif]

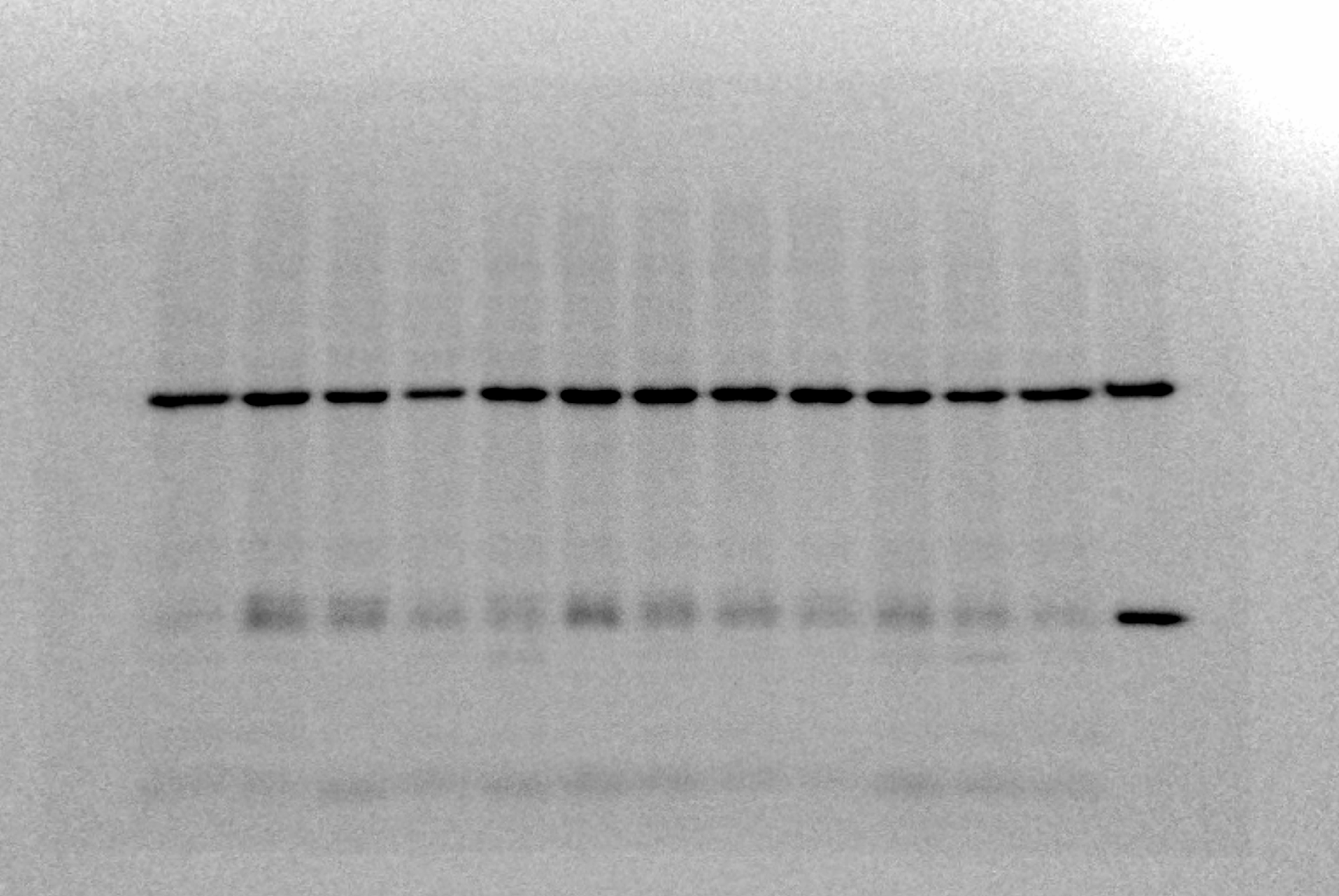

Supplement: Supplementary file 1 [file pharmaceutics-15-00553-s001.zip › Figure S5/48h after drug injection_tubulin_4-6.tif]

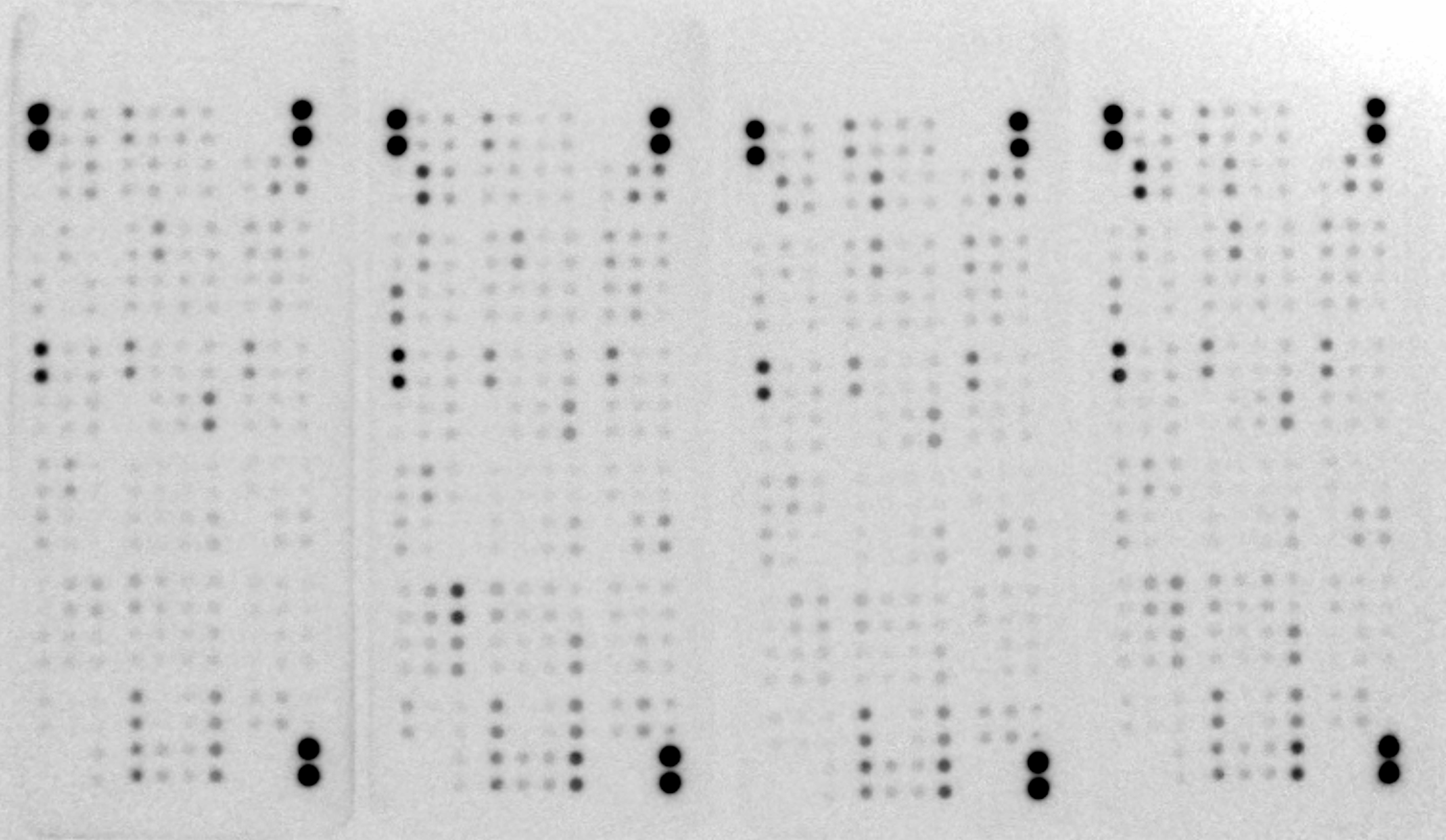

Supplement: Supplementary file 1 [file pharmaceutics-15-00553-s001.zip › Figure S6.tif]

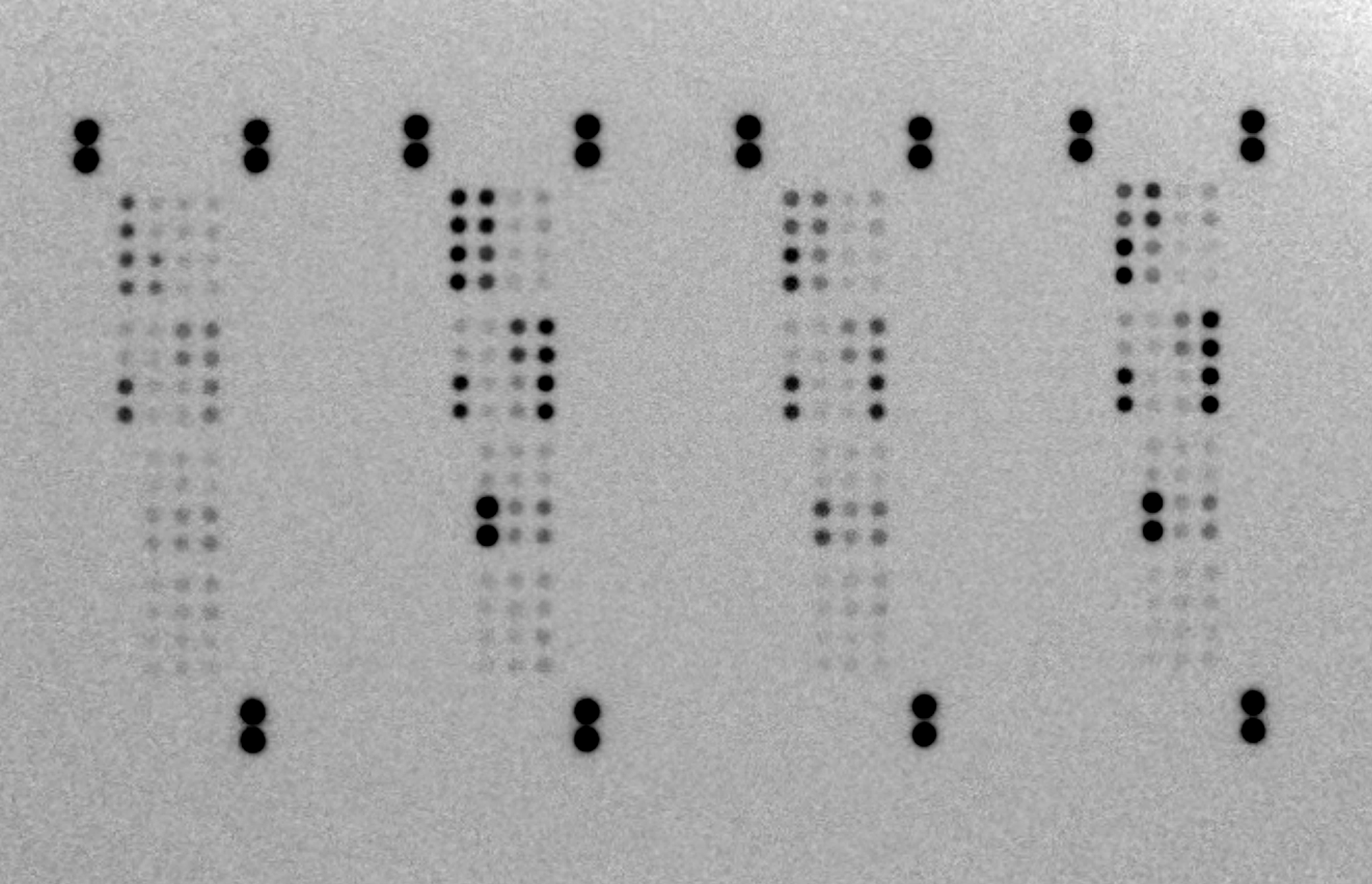

Supplement: Supplementary file 1 [file pharmaceutics-15-00553-s001.zip › Figure S7.tif]
